# Influence of WO_3_-Based Antireflection Coatings on Current Density in Silicon Heterojunction Solar Cells

**DOI:** 10.3390/nano13091550

**Published:** 2023-05-05

**Authors:** Doowon Lee, Myoungsu Chae, Ibtisam Ahmad, Jong-Ryeol Kim, Hee-Dong Kim

**Affiliations:** 1 Department of Semiconductor Systems Engineering, and Convergence Engineering for Intelligent Drone, Institute of Semiconductor and System IC, Sejong University, 209, Neungdong-ro, Gwangjin-gu, Seoul 05006, Republic of Korea; dwlee@sejong.ac.kr (D.L.); saintcms@naver.com (M.C.); imibtisam@outlook.com (I.A.); 2 Department of Optical Engineering, Sejong University, 209, Neungdong-ro, Gwangjin-gu, Seoul 05006, Republic of Korea; jrkim@sejong.ac.kr

**Keywords:** anti-reflection coating, WO_3_, silicon heterojunction solar cell

## Abstract

Antireflection coatings (ARCs) with an indium thin oxide (ITO) layer on silicon heterojunction solar cells (SHJ) have garnered significant attention, which is due to their potential for increasing current density (J_sc_) and enhancing reliability. We propose an additional tungsten trioxide (WO_3_) layer on the ITO/Si structure in this paper in order to raise the J_sc_ and demonstrate the influence on the SHJ solar cell. First, we simulate the J_sc_ characteristics for the proposed WO_3_/ITO/Si structure in order to analyze J_sc_ depending on the thickness of WO_3_ using an OPAL 2 simulator. As a result, the OPAL 2 simulation shows an increase in J_sc_ of 0.65 mA/cm^2^ after the 19 nm WO_3_ deposition on ITO with a doping concentration of 6.1 × 10^20^/cm^2^. We then fabricate the proposed samples and observe an improved efficiency of 0.5% with an increased J_sc_ of 0.75 mA/cm^2^ when using a 20 nm thick WO_3_ layer on the SHJ solar cell. The results indicate that the WO_3_ layer can be a candidate to improve the efficiency of SHJ solar cells with a low fabrication cost.

## 1. Introduction

One of the challenges in the photovoltaics industry is to increase the light conversion efficiency as well as decrease the manufacturing cost of solar cells [1,2,3,4,5,6,7]. The first crystalline silicon (c-Si)-based solar cell was demonstrated at Bell Labs in 1954 [8], and various technologies have been reported in order to address the challenges. In recent years, perovskite solar cells have gained significant attention due to their high efficiency of over 25% [9,10,11,12,13,14,15]. The most common solar technology, c-Si solar cells, accounts for over 90% of the global photovoltaic market due to its high efficiency and low manufacturing cost. Researchers and industry experts are constantly looking for ways to improve the efficiency and lower the manufacturing costs of c-Si solar cells in order to make solar energy a more affordable replacement for fossil fuels. It is critical to first comprehend the fundamental operation of a solar cell in order to comprehend the current research trends for c-Si solar cells. The photovoltaic effect is used by a solar cell to convert solar energy into electrical energy. When sunlight is absorbed by a solar cell, pairs of electrons and holes are formed and move in a specific direction to create an electric current. The proportion of sunlight that is converted into electricity is the efficiency of solar cells. The materials used, the processing techniques used, and the production yield all affect how much it costs to manufacture a solar cell. Over the past few decades, the efficiency of c-Si solar cells has been steadily rising. The Passivated Emitter and Rear Cell (PERC) solar cell was first proposed by the University of New South Wales (UNSW) in 1983 [16]. This type of solar cell features a passivation layer on both the front and rear surfaces of the cell to minimize electron recombination and increase the efficiency of the cell. After several years of research and development, the PERC solar cell achieved the highest efficiency of 22.8% in 1989 [17]. This was a significant milestone in the advancement of solar cell technology and demonstrated the potential of passivation layers to improve solar cell efficiency. Since then, PERC solar cells have continued to evolve and improve, and they are now widely used in the solar industry due to their high efficiency and relatively low cost. One of the efforts to increase the efficiency of solar cells is a buried-contact solar cell. A buried-contact solar cell is a type of solar cell that features buried contact points on the front surface of the cell. This design allows for a more efficient collection of light and reduces shading losses, which can improve the overall efficiency of the solar cell. In a buried-contact solar cell, the contact points are buried in trenches etched into the surface of the cell. This design helps to reduce the amount of metal used in the cell and reduces the reflection of light from the surface of the cell, increasing the amount of light that is absorbed by the cell. Buried-contact solar cells can have a higher efficiency compared to traditional solar cells because they reduce the amount of energy that is lost due to reflection and shading. However, since the theoretical maximum efficiency for single-junction c-Si solar cells is about 29%, achieving even higher efficiencies has become more difficult. As a result, scientists are looking into various strategies to boost c-Si solar cells’ efficiency beyond this threshold.

Utilizing light-trapping structures that can increase the amount of light absorbed by the solar cell is one promising strategy for improving the efficiency of c-Si solar cells. Light-trapping structures are made to scatter or reflect sunlight back into the solar cell, extending the photons’ paths and increasing the likelihood that photons will be absorbed. A solar cell’s textured surface, which can scatter incoming light in various directions and raise the likelihood of absorption, is one example of a light-trapping structure. Plasmonic nanoparticles and photonic crystals are two additional light-capturing structures currently under investigation. Using copper plating is another method to boost the performance of c-Si solar cells. In order to increase the solar cell’s ability to absorb incoming light and thus increase its efficiency, copper plating is used. Copper plating is the process of depositing tiny finger-like lines of metal on the solar cell’s surface. These contacts are crucial for connecting a solar cell’s electrical charge to an external circuit. Because it is highly conductive and less expensive than other metals such as silver or gold, copper is a good choice for this application.

Tunnel oxide passivated contact (TOPCon) solar cells are a type of c-Si solar cell with a carrier-selective contact (CSC) structure that has gained increasing attention due to their excellent surface passivation and carrier extraction characteristics. The CSC structure of TOPCon solar cells includes conductive transport layers or ultra-thin dielectrics that extract one type of carrier (either electrons or holes) from the silicon substrate while effectively passivating the surface of the cell to minimize recombination losses. This selective carrier extraction and surface passivation leads to improved efficiency as well as enhanced lateral carrier transport characteristics that allow for more efficient charge carrier collection and reduced resistive losses [18,19]. Overall, the combination of the CSC structure and the TOPCon technology offers significant potential for improving the performance of c-Si solar cells, with ongoing research focused on optimizing the design and fabrication techniques to further improve their efficiency and reduce their cost. c-Si solar cells with intrinsic a-Si:H (i-a-Si:H) and n+ or p+ a-Si:H, also called silicon heterojunction (SHJ) solar cells, have been successfully demonstrated to achieve a high-efficiency silicon solar cell of 25.6% via an integrated back-contact structure [18]. It achieved a 26.7% efficiency in 2017, the highest efficiency in the crystalline silicon solar cell industry [1]. Technologies can be applied to increase the open-circuit voltage (V_oc_), short-circuit current density (J_sc_), and fill factors (FF) of solar cells to improve their efficiency [20,21,22,23]. SHJ solar cells, which feature insulator and TCO structures instead of TCO, were recently proposed in order to increase J_sc_ [24,25,26,27]. This increase in J_sc_ is explained by the double-layered antireflection coatings (DLARCs) principle. DLARCs are types of anti-reflective coatings (ARCs) that consist of two different layers of material on the surface of a solar cell. This design can increase the efficiency of a solar cell by providing improved anti-reflective properties compared to a single-layer coating. The DLARC principle is to improve J_sc_ by reducing the total reflectance of the solar cell due to the constructive and destructive interference of the reflected light from each layer. A. Cruz et al. used SiO_2_ as DLARC on the top of an ITO in order to decrease the reflectance, which resulted in an increase in J_sc_ to 40.4 mA/cm^2^ [26]. In addition, W. Liu et al. reported that SiO_x_ deposition on the solar cell exhibits improved damp-heat stability in 1000-h aging [28]. SHJ solar cells with an insulator have achieved great high-efficiency c-Si solar cells, but various studies that are related to other insulators for the ARCs should be conducted in order to ensure their feasibility.

**Figure 1:**
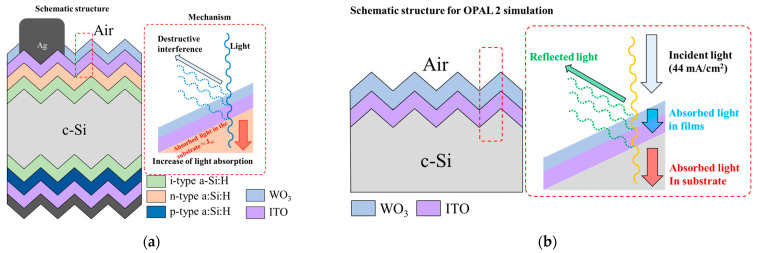
(**a**) Schematic structure of the SHJ solar cell with WO_3_-based ARCs. (**b**) Schematic structure for the OPAL 2 simulation.

The goal of this study was to experimentally evaluate the influence of a WO_3_ layer as an ARC on an SHJ solar cell, which is shown in Figure 1a. The reason for using WO_3_ is that it has high transparency in visible light and is a stable and durable material, which can lead to the stable long-term performance of solar cells [12]. In addition, it can be applied to solar cells such as perovskites and organic solar cells [29,30]. OPAL 2, which uses mathematical models, including the effect of the refractive index as a function of wavelength dependence upon the materials and the thicknesses [31], was used prior to the experiment for the reflectance simulation. A thin WO_3_ layer was deposited on the SHJ solar cell, and the characteristics were analyzed based on the simulator data. As a result, we obtained a current density improvement of 1.25 mA/cm^2^ when 20 nm of WO_3_ was placed on the SHJ solar cell. Compared to other papers using IZO, HfO_2_, and Al_2_O_3_ [22,32,33], we also obtained a current density improvement of about 0.59 mA/cm^2^.

## 2. Materials and Methods

### 2.1. Minimization of the Reflectance Using a Simulation with a WO_3_/ITO/Si Substrate Structure

Figure 1b shows the proposed structure with a WO_3_ layer in order to analyze J_sc_, which is expressed in the OPAL 2 simulation as the absorbed current density in the c-Si substrate (J_sub_) [31]. First, to optimize the ITO thickness, simulations were conducted to maximize the current density according to the doping concentration of ITO in the Air/ITO/c-Si structure. The thickness of the c-Si substrate was set to 180 μm, and the name of the simulation parameter for the c-Si substrate was “crystalline (Gre08), 300 K”. The parameter names for ITO were “Sputtered 2.0 × 10^20^/cm^3^ [Hol13]”, “Sputtered 4.9 × 10^20^/cm^3^ [Hol13]”, and “Sputtered 6.1 × 10^20^/cm^3^ [Hol13]”, where the number in the parameter name means the doping concentration of ITO. These doping concentrations were chosen based on the doping concentrations of ITO typically applied in high-efficiency solar cells [24,34]. The surface morphology of the c-Si substrate was set to random upright pyramids. The angle of the upright pyramid was set to 54.74°, which is typical for solar cells with a surface textured with a tetramethylammonium hydroxide (TMAH) solution [35]. The planar fraction was set to 0%. The light-trapping model was defined as follows:

(1) $$Z=4+\frac{\ln\left(n^{2}+\left(1–n^{2}\right)e^{-4\alpha W}\right)}{\alpha W}$$

where n is the refractive index of the ARC, Z is the optical pathlength, W is the width of the substrate, and α is the polarization angle [36]. The spectrum of the incident illumination was set to a parameter named “AM 1.5 g [Geu95]”, and the zenith angle was set to 0°. The current density when the given incident illumination is absorbed by the substrate without any loss (J_incident_) is 44.0 mA/cm^2^, which is approximately the same as the current density of a solar cell. Therefore, the above current density is defined as the incident light current density. As the incident illumination was injected into the Air/ITO/c-Si structure, the current density of reflected light (J_R_), current density representing all the light absorbed by the film (J_ab_), and finally J_sub_ were analyzed. In detail, the J_R_ in this structure is the amount of total light reflected by the structure. The J_ab_ in this structure is the amount of light absorbed by the ITO expressed as a current density. Therefore, it can be defined as the following equation:

(2) $$J_{\mathrm{incident}}=44.0\;\mathrm{mA}/{\mathrm{cm}}^{2}=J_{R}+J_{\mathrm{ab}}+J_{\mathrm{sub}}$$

The thickness that can maximize J_sub_ according to the doping concentration of ITO was first secured. Next, simulations were conducted with the Air/WO_3_/ITO/c-Si structure shown in Figure 1 based on the optimized thickness depending on the doping concentration of ITO. The parameter of WO_3_ was set to the parameter named “amorphous [Hut06]”, and the thickness that can maximize J_sub_ when WO_3_ is deposited was obtained.

### 2.2. WO_3_ Deposition on an SHJ Solar Cell

N-type Czochralski c-Si wafers with a thickness of 180 μm and a resistivity of 3.8 Ω-cm were used. Both surfaces of the Si wafer were randomly textured. Amorphous i/n and i/p a-Si:H layers were deposited on the front and back surfaces of the c-Si wafer. An 80 nm thick ITO layer was deposited afterward on both sides. The deposited ITO had a sheet resistance of ~90 Ω/sq and an approximate doping concentration of 5.0 × 10^20^/cm^3^ [37]. The front and back contacts were then formed by evaporation and plating after a photolithography process. WO_3_ was subsequently deposited by a thermal evaporator at a deposition rate of 0.1 Å/s on the SHJ solar cell after metallization. The thickness of WO_3_ varied from 5 to 25 nm. The solar cell parameters were measured before and after the WO_3_ deposition using a solar simulator (Wacom Electric Co., Ltd., Fukaya-shi, Saitama, Japan) in order to analyze the solar cell characteristics. The external quantum efficiency (EQE) was measured in order to compare the efficiency of the solar cells. To analyze its influence, the reflectance was measured using UV-VIS spectroscopy (Agilent, Cary5000, Santa Clara, CA, USA). In addition, WO_3_ was also deposited on a polished Si wafer in order to investigate the refractive index via an ellipsometer (J. A Woollam Co., Ltd., Alpha SE, Lincoln, NE, USA). In addition, the surface of the solar cell was analyzed by atomic force measurement (AFM, Park System Corp., Suwon-si, Gyeonggi-do, Republic of Korea) to confirm the surface morphology, and the cross-section of the solar cell was observed by high-resolution transmission electron microscopy (HR-TEM, JEOL, JEM-2100, Akishima, Tokyo, Japan) to confirm that WO_3_ was deposited uniformly. For HR-TEM, the samples were prepared using a focused ion beam (FIB) system (FEI, Quanta 3D FEG, Hillsboro, OR, USA).

## 3. Results

### 3.1. Simulation Results with a WO_3_/ITO/Si Substrate Structure Using an OPAL 2 Simulator

**Figure 2:**
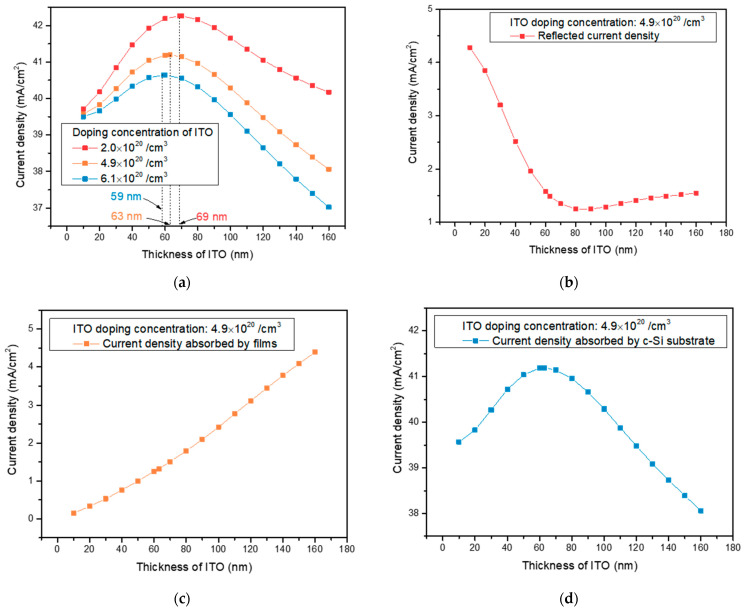
(**a**) J_sub_ as a function of ITO thickness and doping concentration in Air/ITO/c-Si structure. (**b**) J_R_, (**c**) J_abs_, and (**d**) J_sub_ as a function of thickness of ITO in the Air/ITO/c-Si structure when the doping concentration of ITO is 4.9 × 10^20^/cm^3^.

Prior to the simulation with WO_3_, simulations were performed on the Air/ITO/c-Si structure to obtain the optimized thickness as a function of ITO doping concentration. Figure 2a shows J_sub_ as a function of ITO doping concentration and thickness. As a result, when the doping concentrations of ITO were 2.0 × 10^20^, 4.9 × 10^20^, and 6.1 × 10^20^/cm^3^, the thicknesses that could maximize J_sub_ were 69, 63, and 59 nm, respectively. To further analyze the effect of ITO thickness, we analyzed J_R_, J_abs_, and J_sub_ as a function of ITO thickness, representative of a sample with a doping concentration of 4.9 × 10^20^/cm^3^ (Figure 2b–d). In Figure 2b, J_R_ gradually decreased as the thickness of the ITO increased and then started to decrease after the optimized thickness of the ITO. The variation in J_R_ with thickness is due to the change in total reflectance due to the destructive and constructive interferences between the light reflected from the ITO surface and the light reflected between the ITO and c-Si. For J_abs_, as the thickness increased, the absorbed current density continued to increase, as shown in Figure 2c. In the case of J_sub_, as shown in Figure 2d, we observed that it increased as the thickness of the ITO increased and then decreased again when the thickness of the ITO was above the optimum thickness. As the thickness was below the optimum thickness, J_sub_ increased due to the decrease in reflection, but as the thickness increased, absorption in the films increased, and J_sub_ tended to decrease again. Therefore, ITO should be deposited below the optimum thickness because the amount of current density absorbed by the thin film increases when depositing a thickness above the optimum thickness. Additionally, the thickness of the ITO is consistent with the literature [10,38,39].

**Figure 3:**
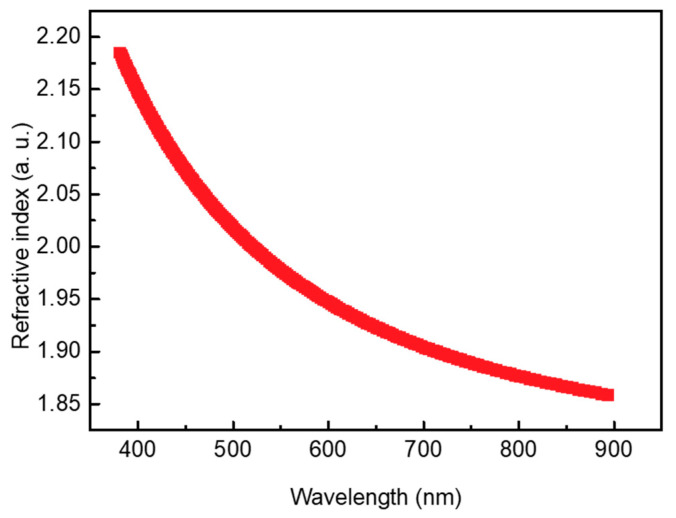
Refractive index of deposited WO_3_ with a thickness of 15 nm.

The refractive index of the deposited WO_3_ was evaluated prior to experimentally investigating the influence of WO_3_ on an SHJ solar cell, as shown in Figure 3. As a result, we observed a refractive index of 1.96 at 580 nm, a relatively low value compared to what was reported in the literature, indicating that the deposited WO_3_ might have some sub-stoichiometric WO_3−x_ phases [40]. WO_3_ was employed in the SHJ solar cell as an ARC.

**Figure 4:**
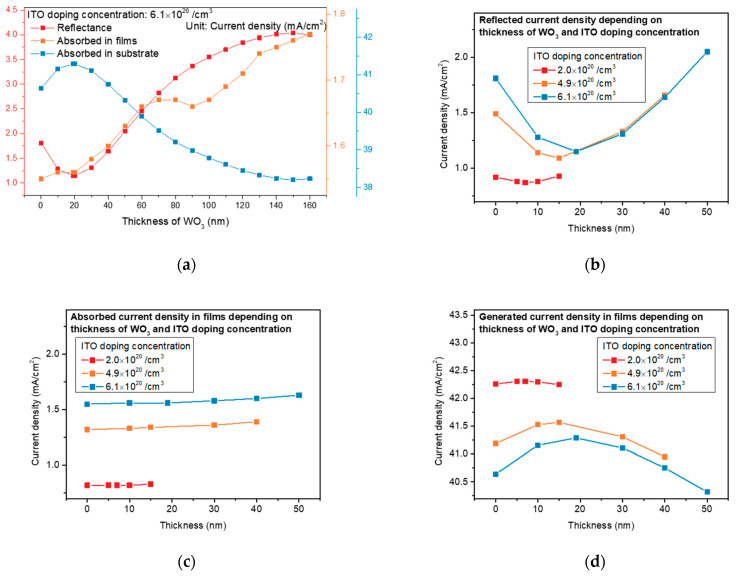
(**a**) J_R_, J_abs_, and J_sub_ depending on WO_3_ as the doping concentration was 6.1 × 10^20^/cm^3^. Simulation results in each current density depending on the WO_3_ thickness and ITO doping concentrations of (**b**) 2.0, (**c**) 4.9, and (**d**) 6.1 × 10^20^/cm^3^.

Before simulating the Air/WO_3_/ITO/Si structure for all ITO doping concentrations, we fixed the doping concentration of the ITO at 6.1 × 10^20^/cm^3^ and evaluated J_R_, J_abs_, and J_sub_ when WO_3_ was continuously increased (Figure 4a). As a result, we observed that J_R_ and J_abs_ continued to increase after the optimized thickness, resulting in a reduction in J_sub_. Based on the above results, we did not perform much simulation beyond the optimized thickness. Figure 4b–d show J_R_, J_ab_, and J_sub_ dependence on the thickness of WO_3_ and the ITO doping concentration. We observe in Figure 4b that J_R_ decreased from 0.92 to 0.87 mA/cm^2^ after the deposition of the 7 nm thick WO_3_ at a doping concentration of 2.0 × 10^20^/cm^3^. J_R_ decreased from 1.49 and 1.81 to 1.09 and 1.15 mA/cm^2^ as WO_3_ was deposited at 15 and 19 nm in regard to the doping concentrations of 4.1 and 6.1 × 10^20^/cm^3^, respectively. J_R_ was affected by the deposition of WO_3_, and the effects were distinctively manifested as the doping concentration of the ITO increased above 4.9 × 10^20^/cm^3^, as shown in Figure 4b. On the other hand, we observe in Figure 4c that the J_ab_ showed minor changes when the thickness of WO_3_ increased for all doping concentrations of the ITO, indicating that the additional WO_3_ deposition had a minor effect on the J_ab_. According to the decrease in J_R_ by the deposition of WO_3_, we observed an improved J_sub_, which is shown in Figure 4d. The J_sub_ for the doping concentration of 2.0 × 10^20^/cm^3^ was slightly elevated due to the minor change in J_R_. We also observed impressive improvement from 40.19 and 40.64 to 41.57 and 41.29 mA/cm^2^ for the doping concentrations of 4.1 and 6.1 × 10^20^/cm^3^, respectively, which was due to the decrease in J_R_, with regard to the high doping concentration above 4.1 × 10^20^/cm^3^. However, as the thickness of WO_3_ increased, the J_sub_ decreased, which was due to the increase in J_R_. This is because the wavelength range for the destructive and constructive interferences was changed by the deposition of WO_3_, which is consistent with the reported literature [24,25,26]. These results indicate that WO_3_ of optimum thickness should be deposited, depending on the ITO doping concentration, to improve the J_sc_, and this should be experimentally demonstrated.

### 3.2. Experimental Results of WO_3_ Deposition on SHJ Solar Cells

**Figure 5:**
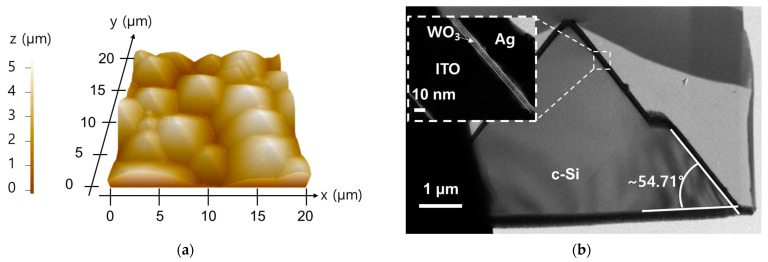
(**a**) AFM and (**b**) HR-TEM results for the SHJ solar cell with 5 nm WO_3_.

First, AFM analysis was performed to analyze the surface morphology of the fabricated solar cells. Figure 5a shows the surface image of a randomly textured solar cell measured by AFM. In order to analyze the uniformity of WO_3_ and the angle of texturing, the cross-section of the solar cell was analyzed by HR-TEM (Figure 5b). As a result, it was found that the surface texture had an angle of about 54.6°, which was similar to the simulation. In addition, it was confirmed that 5 nm of WO_3_ was uniformly deposited.

**Figure 6:**
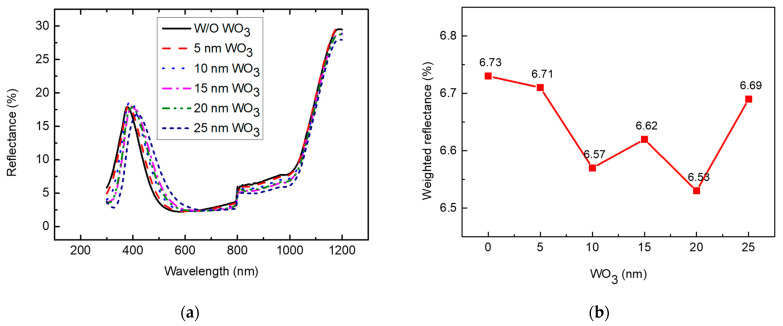
(**a**) UV-VIS results; (**b**) weighted reflectance of SHJ solar cells depending on WO_3_ thickness.

Figure 6a shows the reflectance results after WO_3_ deposition on the SHJ solar cells. The reflectance in the wavelength regions of 200 to 400 nm and 600 to 1200 nm decreased in proportion to the increasing thicknesses of WO_3_. However, the reflectance in the wavelength region of 400 to 600 nm increased with the increasing thickness of WO_3_. This was because the light reflected from the surface of WO_3_, the interface of WO_3_ and the ITO, and the interface between the ITO and the amorphous silicon interfered with each other, which resulted in a change in the wavelength range of the constructive and destructive interference. The minimum reflectance of 2.22% was obtained at 584 nm of light for the sample without WO_3_ deposited on the SHJ solar cell. The minimum reflectance increased as the thickness of WO_3_ increased. The minimum reflectance was 2.42% at a 690 nm wavelength when the thickness of WO_3_ was 25 nm. The reflectance results of the experiment and the simulation showed similar tendencies in regard to the dependence on WO_3_ thickness. To determine the efficiency of a solar cell, both the reflectance and the intensity of sunlight should be considered. The solar-weighted reflectance was calculated using the following equation:

(3) $$\mathrm{Solar}-\mathrm{weighted}\;R=\frac{\int S\left(\lambda \right)\times R\left(\lambda \right)\times \Delta \lambda }{\int S\left(\lambda \right)\times \Delta \lambda }$$

where R(λ) is the %R that was measured and S(λ) is the solar irradiance spectrum (AM1.5G) [41]. According to the calculation, which is provided in Figure 6b, the minimum reflectance increased with an increasing thickness of WO_3_, but the solar-weighted reflectance decreased with WO_3_ deposition. The solar-weighted reflectance decreased to 6.53% when the thickness of WO_3_ was 20 nm. This was due to the decrease in reflectance in the wavelength regions of 200 to 400 nm and 600 to 1200 nm being greater than the increase in reflectance at 400 to 600 nm. These changes in the reflectance in the wavelength ranges are caused by the change in the constructive and destructive interference of the reflected light after WO_3_ deposition.

**Figure 7:**
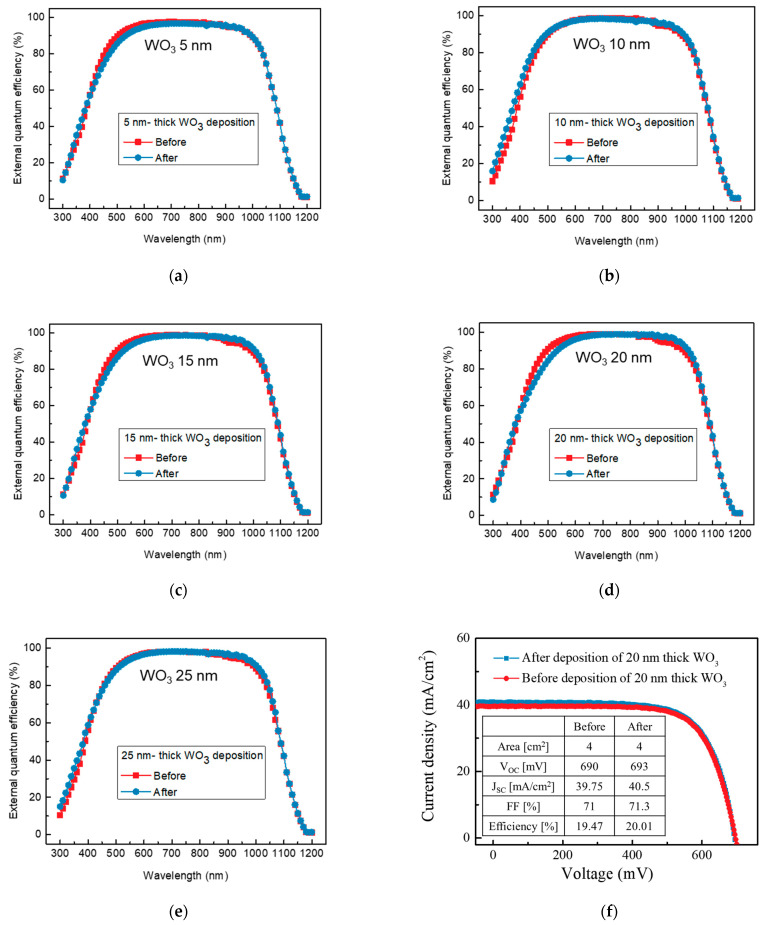
External quantum efficiency (EQE) before and after WO_3_ deposition: (**a**) 5, (**b**) 10, (**c**) 15, (**d**) 20, and (**e**) 25 nm. (**f**) J_sc_–V_oc_ graph before and after the deposition of 20 nm thick WO_3_.

We measured the EQE as shown in Figure 7a–e. According to the change in reflectance, a decrease and increase in each wavelength range were also observed. These results thus show that WO_3_ deposition can improve the J_sc_ of the SHJ solar cell.

A solar simulator was employed to measure the solar cell parameters before and after the deposition of WO_3_ in order to directly investigate the influence of WO_3_ deposition on the SHJ solar cell. Table 1 shows the average measurement result of the solar simulator before and after WO_3_ deposition. There were minor changes after the deposition of WO_3_ for V_oc_ and the fill factor (FF), but no clear trend was observed. However, we commonly observed that the J_sc_ increased after the deposition. J_sc_ was 0.42 mA/cm^2^ higher than the original when the thickness of the WO_3_ layer was 5 nm. The maximum J_sc_ gain was 0.75 mA/cm^2^ when the thickness of WO_3_ was 20 nm, which resulted in an increased efficiency of roughly 0.54%. Figure 7f shows the J_sc_–V_oc_ graph before and after the deposition of 20 nm thick WO_3_, which indicates that J_sc_ increased. The reason for this improvement was similar to the employment of double-layer ARC technology. The decrease in reflectance in the wavelength regions of 200 to 400 nm and 600 to 1200 nm was greater than the decrease at 400 to 600 nm as WO_3_ was deposited, which is shown in Figure 6a. This resulted in improved EQE and J_sc_ after the 20 nm WO_3_ deposition. We thus believe that the proposed WO_3_ can be a springboard that can be used in order to increase efficiency and decrease manufacturing costs.

## 4. Conclusions

The OPAL 2 simulation in this paper was performed on a WO_3_/ITO/Si substrate structure in order to evaluate the influence of WO_3_ deposition on an SHJ solar cell in advance. An optimum condition of WO_3_ was then experimentally introduced on the SHJ solar cell based on the simulation results. As a result, the EQE decreased between 400 and 600 nm, but we observed a rise in the total EQE in the two regions of 200 to 400 nm and the long-wavelength area. The reflectance also showed the same trend as the EQE. The experimental results indicated that the highest J_sc_ was 0.75 mA/cm^2^ when employing 20 nm thick WO_3_ on the SHJ solar cell, which results from the decrease in reflectance due to the effect of the ARCs via WO_3_. The efficiency can therefore be improved by approximately 0.54% by employing an additional WO_3_ layer. We believe that the proposed technique can be a stepping stone to decreasing the levelized cost of energy.

## Figures and Tables

**Table 1:** Results of solar simulator before and after Al_2_O_3_ deposition.

Thickness (nm)	5		10		15		20		25	
	Before	After	Before	After	Before	After	Before	After	Before	After
V_oc_ (mV)	706	708	675	669	697	697	690	693	709	711
J_sc_ (mA/cm^2^)	39.63	40.07	39.84	40.07	40.40	40.28	39.75	40.50	39.86	40.08
FF (%)	75.2	75.9	69.9	67.4	73.1	72.2	71	71.3	76.2	77.3
Efficiency (%)	21.04	21.53	18.8	18.07	20.58	20.27	19.47	20.01	21.53	22.03

## Data Availability

The data presented in this study are available upon request from the corresponding author.
